# High Temperature Effects on Water Loss and Survival Examining the Hardiness of Female Adults of the Spider Beetles, *Mezium affine* and *Gibbium aequinoctiale*

**DOI:** 10.1673/031.009.6801

**Published:** 2009-12-04

**Authors:** Jay A. Yoder, Michael J. Chambers, Justin L. Tank, George D. Keeney

**Affiliations:** ^1^ Department of Biology, Wittenberg University, Ward Street at North Wittenberg Avenue, Springfield, OH 45501 USA; ^2^ Department of Entomology, The Ohio State University, 318 West 12th Avenue, Columbus, OH 43210 USA

**Keywords:** heat shock, water loss, critical transition temperature, activation energy

## Abstract

A remarkable ability to tolerate temperatures as high as 52°C for *Mezium affine* Boieldieu and 56°C for *Gibbium aequinoctiale* Boieldieu (Coleoptera: Anobiidae) was discovered as part of a water balance study that was conducted to determine whether desiccation-resistance (xerophilic water balance classification) is linked to survival at high temperature. Characteristics of the heat shock response were an intermediate, reversible level of injury, appearing as though dead; greater recovery from heat shock by *G. aequinoctiale* (57%) than *M. affine* (30%) that supplemented higher temperature survival by *G. aequinoctiale;* and lack of protection generated by conditioning at sublethal temperature. Heatinduced mortality is attributed to an abrupt, accelerated water loss at 50°C for *M. affine* and 54°C for *G. aequinoctiale*, not to the species (*M. affine*) that loses water the slowest and has the lower activation energy, *E*_a_ as a measure of cuticular boundary effectiveness. These temperatures where water loss increases sharply are not critical transition temperatures because Arrhenius analysis causes them to be erased (uninterrupted Boltzmann function) and *E*_a_ fails to change when cuticular lipid from these beetles is removed. Our conclusion is that the temperature thresholds for survival and accelerated water loss closely match, and the key survival element in hot and dry environments contributing to wide distribution of *G. aequinoctiale* and *M. affine* derives from rising temperature prompting entry into quiescence and a resistance in cuticular lipid fluidity.

## Introduction

One of the most remarkable features of many spider beetles, including the American spider beetle *Mezium affine* Boieldieu and the shiny spider beetle *Gibbium aequinoctiale* Boieldieu (Coleoptera: Anobiidae), is their ability to survive for nearly 3 months without food and water under desiccating conditions of 0% RH and 25°C (Benoit et al. 2005), a capacity that likely contributes to them being one of the most persistent, widely-distributed stored product, warehouse and household pests (Bellés and Halstead 1985). Their impressive ability to thrive under these conditions is due to characteristics of resisting water loss (xerophilic water balance) with an emphasis on water conservation as adults, featuring impermeable cuticle, low percentage body water content and low net transpiration rate (Benoit et al. 2005). This is supplemented behaviorally by capacity for quiescence (Howe 1959) and morphologically by a covering of body hair (insulation), fusion of the elytra, and reduced or absent metathoracic wings giving them their unique globular body shape resembling a spider (Philips 2000). In those that can woodbore, a notable sign of infestation is tunneling/destruction in wood or other material by larvae searching for a site to pupate. Adults emerge, act as scavengers, and are mostly active at night, averaging a period of 1 month to change from egg to adult. Beetle frass and silk from cocoons are other signs of infestation (Howe 1959). These beetles can also be found as nest associates of rodents, birds and bats where they primarily feed on their feces (Howe 1959; Bellés and Halstead 1985). Particularly problematic is the prevalence of spider beetles as domestic pests that can be found in dried baby food, dog biscuits, woolens, towels, leather and paste, and they show a common co-occurrence with rats (Gross 1948). Old and out of condition rodent baits from pelletized bait placements in the basement of a building in downtown Columbus, OH was the origin of some of the spider beetles (*M. affine*, *G. aequinoctiale*) used in this study. Whether the extreme desiccation tolerance of spider beetles translates into other hardiness features of their biology is not known.

Rising temperature has a profound impact on water loss by promoting desiccation (Hadley 1994), suggesting that the resistance to water loss in spider beetles may contribute to their ability to maintain adequate levels of body water necessary to function properly at high temperature. To test this hypothesis, a combined survivorship and water loss experiment with an emphasis on cuticular lipid, the water proofing barrier known to protect against desiccation (Blomquist et al. 1987). We explored possible correlations between heat stress and desiccation resistance. The focus of this study was on the temperature at which cuticular lipids undergo a phase change known as the critical transition temperature. This temperature is signified by a change in activation energy, *E*_a_ (Gibbs 2002), as a measure of cuticular lipid effectiveness to restrict water loss (Yoder et al. 2005). When an arthropod reaches and exceeds the critical transition temperature, there is a rapid acceleration in water loss rate (Wharton 1985) that conceivably leads to a lethal level of desiccation. Therefore, the greater the extent of water proofing (due to increased quantity or quality of particular cuticular lipid components; Rourke and Gibbs 1999), the higher the temperature required to elicit a change in *E*_a_ corresponding to the phase transition. Thus, high critical transition temperature either reflects greater impermeability of the cuticle or a cuticular modification that enhances water retention and restricts water loss. This is highly applicable to spider beetles because their fused elytra (wingless) designfavors water conservation by eliminating water loss from the wings (Philips 2000; Benoit et al. 2005). Behavioral assessments were used to determine the range of temperatures where *M. affine* and *G. aequinoctiale* can function most effectively and measure water loss rates at increasing temperature (live, dead, delipidized beetles) to determine whether survival is connected to the critical transition temperature.

## Materials and Methods

### Beetles and test conditions

All beetles were reared in 1000 cc glass jars on a consistent diet of rolled oats and cornmeal, held at 15:9 L:D and 25° C and with no free water for many years. The exact age of beetles was not known. An aspirator was used for handling the beetles. For experiments, temperature was controlled by programmable incubators (Fisher Scientific, www.fishersci.com) and varied less than 0.5°C. Calcium sulfate (Drierite, CaSO4) generated 0% RH (Toolson 1978) and glycerol-water mixtures (Johnson 1940) and saturated salt solutions (Winston and Bates 1960) generated 97% RH in sealed glass desiccators (8000 cc) (Thomas Scientific, www.thomassci.com); relative humidity varied less than 1%). One cc mesh-covered chambers were used to store beetles (1 beetle/chamber to permit individual monitoring) without food and water and were placed on a perforated porcelain plate within the desiccators. Weighing was conducted with an electrobalance (± 0.2 μg SD precision and ± 6 μg accuracy at lmg; Cahn G-2); no enclosure or anesthesia was used and beetles were out of test conditions for less than 1 minute for mass determinations. Beetles were used for an experiment after having lost 4–6% body mass (0% RH, 25°C) so that mass measurements reflect changes in body water levels of the beetle and were not obscured by removal of passively adsorbed water or changes due to digestion, excretion or reproduction (Arlian and Ekstrand 1975).

### Determination of critical transition temperature

Beetles were weighed, placed at 0% RH at a test temperature, and then reweighed at various time intervals such that there were six mass measurements (not including initial mass) per temperature. At 0% RH water loss is exponential allowing water loss rate to be derived from the slope of a linear regression (Wharton 1985). Water loss rate for each beetle was based on a total of six mass measurements over time making sure that there was more water to lose i.e., had not achieved dry mass. At the end of an experiment, beetles were placed in a 90°C, 0% RH, to obtain dry mass (*d*) and weighed daily until mass remained constant for three consecutive days. The dry mass was subtracted from each of the six mass measurements to convert each mass measurement into a water mass (*m*) value that represents the amount of water in the beetle. The water loss rate for each individual beetle per temperature was derived from the slope of a regression on a plot as described by the equation *m_t_ = m_0_e^-kt^* or ln *m_t_/m_0_* = -*kt*, where *mt* is the water mass any time *t* and *m_0_* is the initial water mass (Wharton 1985). Percentage body water content was calculated by expressing water mass (*m*) as a proportion of initial mass (fresh mass, *f*) as described: percentage *m* = 100 (*f– d*)/*f*(Wharton 1985).

Critical transition temperature was based on a change in activation energy, *E_a_* (Rourke and Gibbs 1999) as described by the Arrhenius equation: *k* = *^Ae-Ea/(RgasT)^* or ln *k* = *-E_a_*/(*R_gas_T*) + ln *A*, where *k* is water loss rate, *E_a_* is activation energy, *R* is universal gas constant, *T* is absolute temperature and *A* is frequency (steric) factor (Wharton, 1985); slope of the regression is *-E_a_/R*. To conform to standard practice (Seethaler et al. 1979.), freshly killed beetles were used (-20 °C freeze/thaw at 25°C or closed system of HCN vapor) to remove respiratory effects. Living beetles and killed beetles that had their cuticular lipids removed by extraction in 2:lv/v chloroform:methanol, twice for 1 minute, were examined for comparison. Critical transition temperature was based on water loss rates determined for a separate batch of beetles at each temperature and also for the same batch of beetles in a ramp up (transfer from lowest to highest temperature) and ramp down (transfer from highest to lowest temperature) technique.

### Determination of heat tolerance

Beetles were weighed, placed into 1 cc mesh-covered vials (1 beetle per chamber to permit individual monitoring and to eliminate potential group effects), and then exposed to a particular high temperature for 1 hour. Following the 1 hour heat shock, beetles were transferred to 25°C for recovery. Treatment and recovery were held constant at 97% RH. Each beetle was examined microscopically (40x) and scored based on their ability to complete a series of behavioral tasks: 0 = 'active', alive, moving, displays regular, rapid ambulatory activity, ability to right and crawl six body lengths when prodded; 1 = 'injured', alive, slight twitching movement of legs when prodded, legs partially curled, unable to right, dragging hind legs while attempting to crawl and back legs slightly curled; and 2 = 'dead', immobile, lack of movement when prodded, no twitching of legs, unable to right and crawl and unresponsive. These methods represent a combination of those used by Huey et al. (1992), Neargarder et al. (2003), Edgerly et al. (2005) and Yoder et al. (2006). The beetles were then placed back into 25°C and observed after 24 hours undergoing the same examination of behavioral tasks and receiving the appropriate score (0 = active, 1 = injured, 2 = dead). After the 24 hour observation, beetles were dried in the drying oven (90°C, 0% RH) to determine the dry mass (*d*) for calculation of water mass (*m*) and percentage body water content as an assessment of body size.

### Sample sizes and statistics

An analysis of variance (ANOVA) was used to compare data. Data are the mean ± SE of 30 individuals each: 10 beetles per replicate, *n* = 3, with each replicate coming from a different rearing batch. Thus, each temperature in the heat tolerance study consisted of observations on a total n = 30 beetles, with each being examined individually resulting in a total of 180 individual water loss rates (n = 30 at each of six temperatures plus five mass measurement per individual beetle) for each of the critical transition temperature determinations. Percentage data were arcsin transformed prior to analysis. The slopes of regressions (derivation of *E*_a_ and water loss rate, *k*) were based on a test of equality of slopes of several regressions (Sokal and Rohlf 1995). The number of beetles observed in a particular condition was multiplied by its corresponding point value (0 = active, 1 = injured, 2 = dead) to calculate a total number of points that was then expressed as a percentage (conversion of 1.67 because maximum score is 60 for sample of 30 dead beetles) and used as the damage score in the heat tolerance experiment.

## Results

### Beetle size

Percentage body water content of *M. affine* was 63.97 ± 2.2%; water mass, *m* = 3.64 ± 0.18mg; initial (fresh) mass,*f*= 5.69 ± 0.11mg (mean ± SE); water mass to dry mass ratio, *m*trch*/d* = 1.78, for the heat tolerance experiment. Corresponding values for *M. affine* in the critical transition temperature experiments were: 64.58 ± 1.6%, *m* = 3.50 ± 0.23mg;*f* = 5.42 ± 0.07mg; *m/d* = 1.82 for beetles that were tested living; 63.70 ± 2.5%, *m* = 3.51 ± 0.14mg;*f* = 5.51 ± 0.12mg; *m/d* =1.76 for beetles that were tested killed; and 64.94 ± 2.0%, *m* = 3.63 ± 0.14mg;*f*= 5.59 ± 0.11mg; *m/d* = 1.85 for beetles that were tested delipidized. These water balance characteristics were not significantly different across the experimental groups (ANOVA; *p* > 0.05). In all cases, water mass was a positive correlate of dry mass (*r*^2^ ≥ 0.96 for beetles used in the heat tolerance study; *r*^2^ ≥ 0.93 for beetles that were used in the water loss study that were living; *r*^2^ ≥ 0.95 for beetles used in the water loss study that had been killed; *r*^2^ ≥ 0.94 for beetles used in the water loss study that had been delipidized; ANOVA; *p* < 0.001). All *M. affine* were similar in size, shape and water content in the experiment.

*G. aequinoctiale* had a percentage body water content of 61.05 ± 1.8% (*m* = 2.68 ± 0.33;/= 4.39 ± 0.26; *m/d* = 1.57) in the heat tolerance study (correlation of water mass to dry mass, *r*^2^ ≥ 0.94; ANOVA;*p*) < 0.001). These values were not significantly different (ANOVA; *p* > 0.05) from the *G. aequinoctiale* that were used in critical transition temperature experiment: live group (61.43 ± 2.3%; *m* = 2.74 ± 0.27;*f*= 4.46 ± 0.31; *m/d*= 1.59; *m* to *d* correlation, *r^2^* ≥ 0.97); killed group (60.77 ± 1.9%; *m* = 2.68 ± 0.21;*f*= 4.41 ± 0.32; *m/d* = 1.55; *m* to *d* correlation, *r^2^* ≥ 0.96) or delipidized group (61.31 ± 2.4% (*m* = 2.79 ± 0.26;*f*= 4.55 ± 0.22; *m/d* = 1.59; *m* to *d* correlation, *r^2^* ≥ 0.94). Size and shape and water content were similar for *G. aequinoctiale* across the treatment groups. Percentage water content, fresh mass and water mass were significantly different between *M. affine* and *G. aequinoctiale* (ANOVA; *p* < 0.05). Water loss rate at 25°C for living *G. aequinoctiale* was 0.018 ± 0.007%/h which was significantly different from 0.010 ± 0.003%/h for living *M. affine* (ANOVA; *p* < 0.05). Our conclusion is that *G. aequinoctiale* is smaller in size and has less body water and retains water less effectively than *M. affine* and this was consistent across treatment groups, indicating that the differences that noted are species-specific.

### Heat tolerance experiment

**Figure 1.**
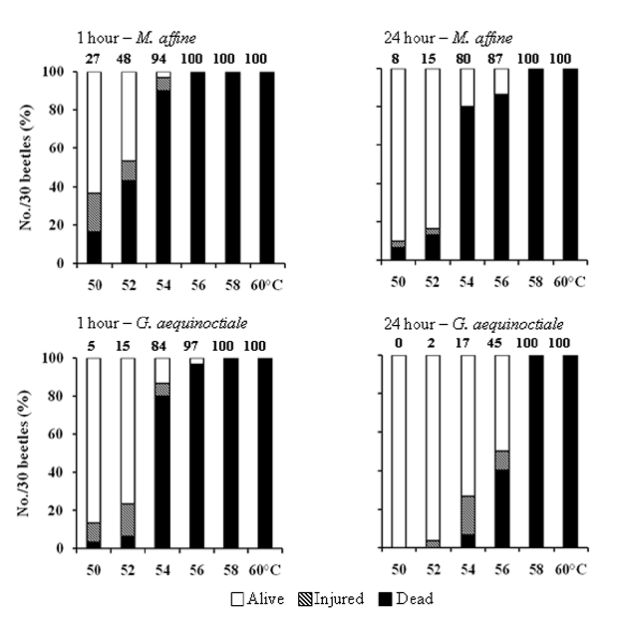
Ability to function properly following 1 hour temperature exposure (constant 97% RH) in female adult *Mezium affine* and *Gibbium aequinoctiale*. Microscopic (40x) observations were made to score the beetles (0 = alive, 1 = injured, 2 = dead), at 1 hour and 24 hour after the I hour heat shock; each temperature represents a total *n* = 30 beetles each (mean ± SE ≤ 2.9). Value above each bar is the damage score based on number of points earned expressed as a percentage.

All 30 *M. affine* (30/30, 100%) displayed regular crawling activity and ability to right at 1 hour and 24 hour observation after 1 hour exposure at 10, 20, 30, 40 and 48°C, and all beetles were killed (30/30, 100%) by 1 hour treatment at 58, 60 and 70°C, but not in the 50 – 56°C range (Figure 1). Complete survival and lack of injury observed following treatment at 10, 20, 30, 40 and 48°C (total *n* = 150 beetles combined) is a useful control showing that the beetles were not unusually sensitive to manipulation in the experiment and results obtained after experiencing the higher temperatures were due to heat shock and not to effects of over-handling. There was no desiccation effect because relative humidity was held constant and nearly saturated throughout at 97% RH. *M. affine* was capable of surviving 1 hour treatment at 52°C with 47% of the beetles surviving after 1 hour, which increased to 83% after 24 hours (Figure 1). One of the most unusual observations was that heat shock was sufficient to cause some of the beetles to be completely immobile and unresponsive to stimuli such that they were counted as dead at the 1 hour observation. However, 24 hours later these beetles were alive, thriving and showed no signs of observable injury; it is important to note that the same criteria were met for dead individuals that were counted dead and actually remained dead the next day. This caused a decrease in damage score from 1 hour to 24 hour observation at 50, 52, 54 and 56°C; the most pronounced recovery effects were noted at 50°C treatment (27% to 8%) and 52°C (48% to 15%). Mortality increased after 24 hours when heat shock was compared at 52 and 54°C from 13% to 80% (largest jump), indicating that *M. affine* can handle up to 52°C (< 50% survival by beetles) heat shock.

The observable change in condition for *M. affine* at the range of 50 – 56°C heat shock temperatures was similar for *G. aequinoctiale* (Figure 1); there was 100% mortality (30/30, no survivors after 24 hours) following 1 hour treatment at 58 and 6O°C and 100% living (30/30) after 1 hour treatments at 10, 20, 30, 40 and 48°C (each *n* = 30). Like *M. affine, G. aequinoctiale* showed no mortality and injury (n = 150 beetles) at 10, 20, 30, 40 and 48°C, indicating that they were not sensitive to handling in the experiment. Similarly, a 1 hour treatment within range of 50 – 56°C and assessment by behavioral criteria caused some beetles to be counted as dead at 1 hour after treatment that were subsequently found as alive at 24 hours after treatment, showing that they display intermediate levels of injury. The greatest recovery for *G. aequinoctiale* was noted after treatment at 54°C with 80% counted as dead 1 hour after treatment that decreased to 7% dead when they were observed 24 hours later (decrease in damage score from 84% to 17%). A less pronounced recovery for *G. aequinoctiale* was noted for 1 hour and 24 hour observations after treatment at 56°C (Figure 1), with 97% to 40% mortality and 97% to 45% damage score (ANOVA; *p* < 0.05). By comparison, the greatest recovery response for *M. affine* was noted at 52 and 54°C and the majority of *M. affine* were killed by 1 hour heat shock at 54 and 56°C (Figure 1); temperature range is higher for recovery (54 – 56°C) by *G. aequinoctiale* (ANOVA; *p* < 0.05). Our conclusion is that *G. aequinoctiale* handles 56°C (survival by > 50% of beetles) and has greater ability to recover from heat shock than *M. affine*.

Greater tolerance in some insects can be generated by a brief exposure to a sublethal temperature that generates protection against higher, lethal temperature injury (Lee and Denlinger 1991). Using the same experimental design (1 hour exposure to 50 – 60°C by 2°C increments, 40x microscopic observation 1 hour and 24 hours later), sample size (n = 30 beetles per temperature for *G. aequinoctiale* and *M. affine*) and living beetles that had been previously exposed for 1 hour at 52°C (*M. affine*) and 54°C (*G. aequinoctiale*) and used a day later, we obtained results that compared favorably, showing no statistical difference (ANOVA; *p* > 0.05), with Figure 1 (data not shown). Thus, *G. aequinoctiale* and *M. affine* seem to lack a rapid resistance mechanism to cope with high temperature.

### Water loss experiment

**Figure 2.**
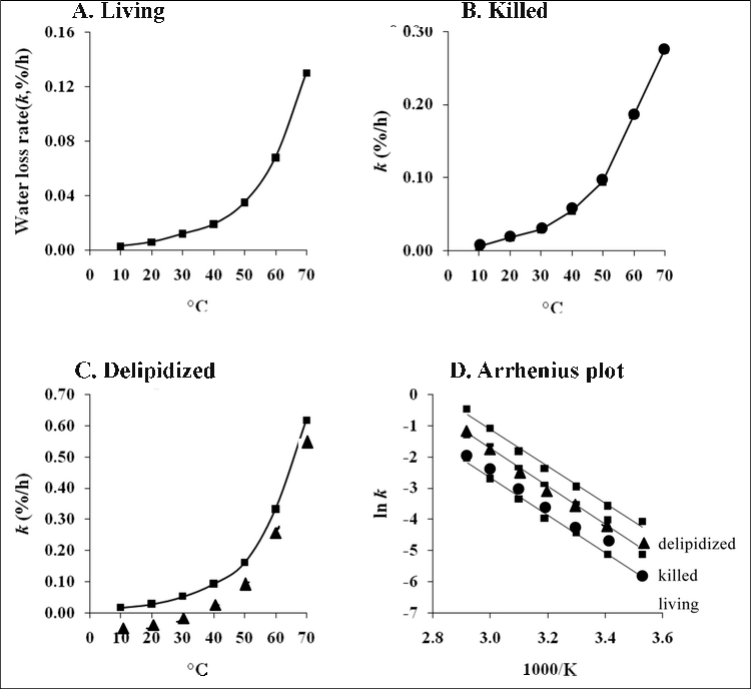
Water loss as a function of temperature in female adults of *Mezium affine* on a linear plot before (A, living; B, killed) and after cuticular lipid extraction (C, delipidized); D, same data re-plotted on an Arrhenius plot to determine activation energy, *E*_a_ (slope of regression = -*E_a_*/*R_gas_*). HCN-killed beetles produced nearly identical results and results were similar for ramp up and ramp down experiments, *n*= 30 beetles for each individual point on the graph (± SE ≤ 0.06). *Gibbium aequinoctiale* yielded similar results except the entire relationship was shifted up the ordinate to reflect their higher water loss rate and the inflection occurred at 54° C rather than at 50°C for *M. affine* (data not presented).

Increasing temperature resulted in an increase in water loss and displayed an exponential pattern on a linear plot for *M. affine* (Figure 2A–C). Compared to living beetles (Figure 2A), water loss rates were 2 – 3x higher for killed beetles (Figure 2B), showing the importance of life processes for controlling respiratory water loss, and water loss rates were 3 – 5x higher for beetles that had been delipidized (Figure 2C), which demonstrates the importance of cuticular lipids at restricting water loss (ANOVA; *p* < 0.05). Consistently, the inflection in the curvature describing water loss for *M. affine* occurred approximately at 50°C where water loss rates increased sharply; noteworthy is the steep increase in water loss at temperatures above 50°C (Figure 2). Nearly identical trends and results were observed whether water loss measurements were conducted on the same group of beetles that was transferred from 10 to 70°C (ramp up), 70 to 10°C (ramp down) or when an individual group of beetles was examined at each temperature separately (data not shown). Curvature for living, killed and delipidized *M. affine* (Figure 2A–C) becomes nearly a straight line in typical Boltzmann fashion (*r*^2^ > 0.99; ANOVA; *p* < 0.001) on an Arrhenius plot that is used to derive activation energy (*E*_a_) (Figure 2D); *E*_a_ values for *M. affine* were 50.1kJ when living, 50.6kJ when killed and 48.9kJ when delipidized and are not significantly different from each other (ANOVA; *p* > 0.05). Thus, *M. affine* loses water abruptly once temperature exceeds 50°C.

Activation energy for *G. aequinoctiale* was 73.5kJ for living beetles and this was not significantly different from activation energy of 72.7kJ for killed beetles and 74.2kJ for beetles that had been delipidized (ANOVA; *p* > 0.05). Activation energies for *G. aequinoctiale* were higher than *E*_a_ values for *M. affine* (ANOVA; *p* < 0.05) as a product of higher water loss rate of *G. aequinoctiale*. The pattern of water loss for *G. aquinoctiale* was similarly exponential on a linear plot as that exhibited by *M. affine* (Figure 2A–C) showing a similar 2 – 3x rate increase as a result of killing and 3 – 5x rate increase as a result of delipidizing. However, water loss rate in *G. aequinoctiale* was higher than in *M. affine* resulting in a change in scale (increase on *y*-axis) on the ordinate when living, killed and delipidized beetles were compared. The inflection in the curvature that took place for *G. aequinoctiale* at around 54°C straightened out (*r* ^2^ > 0.99; *p* < 0.001) by Arrhenius analysis (data not shown), giving rise to single *E*_a_s. Similar to *M. affine*, results for *G. aequinoctiale* in ramp up, ramp down and individual groups testing each temperature separately were alike (data not shown). We concluded that at 54°, water loss accelerates rapidly for *G. aequinoctiale* and features higher activation energies than *M. affine*.

## Discussion

Ability to tolerate 52°c by *M. affine* and 56° C by *G. aequinoctiale* is an impressive feat considering the small body size of these beetles and is a biologically important feature. The fact these temperatures correspond to the temperature where water loss rate accelerates rapidly is especially noteworthy suggesting that the temperature threshold of survival and the temperature threshold of a particularly rapid water loss are linked. The ability of these spider beetles to tolerate high temperature is not linked to the species that loses water the slowest (i.e., with the lower net transpiration rate, *M. affine*), but rather to the species that can retain water more effectively until higher temperatures are reached (i.e., resists abrupt water loss increase at lower temperature; *G. aequinoctiale*). Absence of a critical transition temperature in *M. affine* and *G. aequinoctiale* implies that part of their ability to tolerate high temperature extremes is because they resist a change in cuticular lipid fluidity that favors water conservation; i.e., there is no change in *E*_a_, indicative of a phase change in cuticular lipid. Like most insects, the heat shock response by *M. affine* and *G. aequinoctiale* was characterized by an intermediate living condition and was not 'all or nothing' (alive or dead; Edgerly et al. 2005) in that they were able to overcome the trauma due to heat shock and regain regular ambulatory activity. Most *M. affine* and *G. aequinoctiale* in this intermediate injury condition were initially counted as dead. Under desiccation stress this behavior can promote water conservation by causing a metabolic shutdown (Benoit et al. 2005). Apparently, heat stress similarly prompts *M. affine* and *G. aequinoctiale* to enter into an immobile quiescent state, presumably enhancing the ability to remain viable.

Consistently, *G. aequinoctiale* was less affected by heat stress than *M. affine* as seen via decreased number of beetles killed at high temperatures and increased recovery from heat shock. *G. aequinoctiale* inhabit microhabitats that are as hot and dry as does *M. affine* and are spread by commerce throughout the world (Bellés and Halstead 1985; Philips 2000), indicating that their water balance characteristics are quite similar (classified as xerophilic), except that the larger *M. affine* loses water more slowly than the smaller *G. aequinoctiale* this most likely being a function of surface area to volume properties (Benoit et al. 2005). Thus, greater heat resistance properties displayed by *G. aequinoctiale* does not appear to be reflected by their distribution and may represent a trade-off with their faster water loss rate for their ability to thrive in a particular habitat and, in fact, may be more microhabitat-related (TK Philips, personal communication, Western Kentucky University, Bowling Green, KY). We conclude that survivorship by *M. affine* and *G. aequinoctiale* at high temperature is due to attributes of: 1. the capacity to retain water effectively (low water loss rate; xerophilic classification; Benoit et al. 2005); 2. their high quantity of dry mass and heavy cuticular sclerotization (low percentage body water content; requires less body water to function; Hadley 1994); 3. the capacity to enter into a heat-induced coma; and, perhaps, 4. having fused elytra and a globular shape that reduces surface area to volume ratio (Philips 2000).

An exponential pattern of water loss is typical in response to increasing temperature, described as 'evaporation curves' in the older literature (Beament 1959), and this pattern is independent of respiratory control and presence of cuticular lipids. The effect is simply a water loss rate increase by 2 – 3 fold as a consequence of killing (spiracular closing mechanisms are inoperable; Hadley 1994) and 3 – 5 fold as a consequence of cuticular lipid removal (no water-proofing; Blomquist et al. 1987), but the overall curvature pattern remains unaltered. Thus, the entire temperature-relationship comparing living, killed and delipidized beetles is shifted up the ordinate in response to increases in water loss by killing and removal of cuticular lipid, such that what changes is the frequency (steric) factor, *A* (*y*-intercept) on the Arrhenius plot as a measure of extent to which killing and/or delipidizing treatment promoted diffusion of water through cuticle (Yoder et al. 2005). Killing and delipidizing have no effect on *E*_a_ compared to living beetles as evidence by similarity of slope (-*E*_a_/*R* on the Arrhenius plot) and reflects proportionate water loss from one temperature to the next. Therefore, the fact that *E*_a_s are higher for *G. aequinoctiale* than *M. affine* indicates that *G. aequinoctiale* experience greater proportionate water loss from one temperature to the next (steeper slope) because their net transpiration (water loss) rate is higher than *M. affine* (Benoit et al., 2005). The difference in *E*_a_s that are noted between *M. affine* and *G. aequinoctiale* is a simple indicator that *M. affine* retains water more effectively than *G. aequinoctiale* and permeability for *M. affine* is less.

It is important to emphasize that according to standard reaction energetics (Glasstone and Lewis 1960), as *E*_a_ increases, the rate of reaction (*k*) decreases (*k* = rate of water loss for our purposes; Wharton 1985). In insects, however, the trend is that *E*_a_s are lowest for those insects that lose water the slowest (again demonstrated here with *M. affine* that loses water slower and has corresponding lower *E*_a_s than *G. aequinoctiale*), suggesting that assessing water loss rate with *E*_a_ in spider beetles, like in other arthropods, is probably an improper designation (Yoder et al. 2005). From a water balance perspective, *E*_a_ reflects the cuticle as a barrier indicative of the amount of energy that is needed by a molecule of body water to cross (Wharton 1985). Activation energy in arthropods does not support a process that is rate-limiting and fails to respond to an artificial water loss rate increase by cuticular lipid removal in that *E*_a_ remains unchanged (similar slope) whether the cuticular lipid barrier is present or not (this study; Yoder et al. 2005). The sharp increase in water loss rate that is observed at rising temperatures approaching the maximum tolerable temperature in *M. affine* and *G. aequinoctiale* is not due to a phase change, 'melting', of cuticular lipid, rather the results indicate that their ability to resist such a transition likely contributes to their persistence.

## Abbreviations

**E_a_**: activation energy
